# Functional repeat-derived RNAs often originate from retrotransposon-propagated ncRNAs

**DOI:** 10.1002/wrna.1243

**Published:** 2014-07-09

**Authors:** Katarzyna Matylla-Kulinska, Hakim Tafer, Adam Weiss, Renée Schroeder

**Affiliations:** Department of Biochemistry and Cell Biology, Max F. Perutz Laboratories, University of ViennaVienna, Austria

## Abstract

The human genome is scattered with repetitive sequences, and the ENCODE project revealed that 60–70% of the genomic DNA is transcribed into RNA. As a consequence, the human transcriptome contains a large portion of repeat-derived RNAs (repRNAs). Here, we present a hypothesis for the evolution of novel functional repeat-derived RNAs from non-coding RNAs (ncRNAs) by retrotransposition. Upon amplification, the ncRNAs can diversify in sequence and subsequently evolve new activities, which can result in novel functions. Non-coding transcripts derived from highly repetitive regions can therefore serve as a reservoir for the evolution of novel functional RNAs. We base our hypothetical model on observations reported for short interspersed nuclear elements derived from 7SL RNA and tRNAs, *α* satellites derived from snoRNAs and SL RNAs derived from U1 small nuclear RNA. Furthermore, we present novel putative human repeat-derived ncRNAs obtained by the comparison of the Dfam and Rfam databases, as well as several examples in other species. We hypothesize that novel functional ncRNAs can derive also from other repetitive regions and propose Genomic SELEX as a tool for their identification.

## THE REPETITIVE GENOME

**Figure 1:**
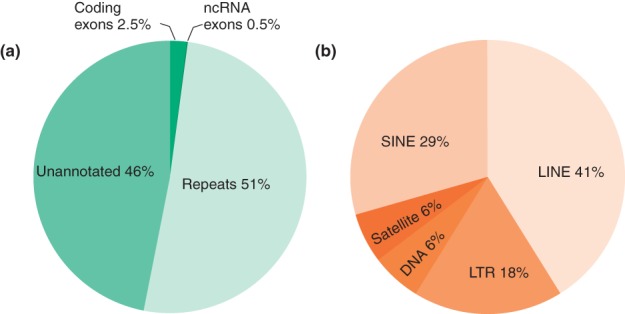
Human genome is repetitive. (a) Composition of the human genome. 2.5 and 0.5% of the human genome is covered with coding exons and non-coding RNA (ncRNA) exons, respectively. Repeats represent 51% of the genome while the unannotated regions amount to 46% of the genome. (b) Composition of the repetitive portion of the human genome. Repeats with the largest genome coverage are long interspersed nuclear elements (LINEs) (41%), followed by short interspersed nuclear elements (SINEs) (29%), long terminal repeats (LTRs) (18%), DNA transposons (6%), and satellite repeats (6%).

The human genome is composed of approximately 3.3 billion base pairs. Canonical genes occupy 30%, but only an estimated 1.5% of the genomic content has protein-coding capacity. Repeats make up at least 51% of the genome^1^,^2^ (Figure 1) and can be classified by sequence similarity, dispersal patterns or by function. Most of the repetitive DNA consists of interspersed transposable elements (TEs), often referred to as parasitic DNA. About 45% of the human genome falls into this class and even more is proposed to be transposon-derived.^2^

TEs are either DNA transposons, which are mobilized by a cut-and-paste mechanism, or retrotransposons, which propagate in the host genome via RNA intermediates in a copy-and-paste manner. Retrotransposons constitute a large fraction of DNA in many eukaryotes, and some of them are still actively retrotransposing, e.g., Alu's germline transposition rate is estimated as 1 per 20 births.^3^ There are three types of mammalian retrotransposons: (1) long interspersed nuclear elements (LINEs) that transpose autonomously and account for 20.4% of the genomic sequence; (2) short interspersed nuclear elements (SINEs) that make up 13.1% of the genome, and their transposition depends on other TEs, such as LINEs, as they lack a functional reverse transcriptase (RT); (3) long terminal repeats (LTRs) that account for 8.3% of the human genome.

Although transposition events can cause damage to the host, there is also substantial evidence that TEs have been important for the evolution and function of genes and genomes.^4^–^7^ It has been suggested that mobile DNA can serve as a dynamic reservoir for new cellular functions because TEs can evolve new genes that are beneficial to the host.^8^ In an analogous way, small RNA-derived retroelements can also give rise to novel RNA-coding genes. The primate BC200 non-coding RNA (ncRNA) is the first known example of an Alu element that evolved into a novel functional small RNA-coding gene.^9^

Another class of genomic repetitive sequences consists of arrays of high-copy-number tandem repeats known as satellite DNA. It accounts for about 8% of the human genome^10^ and is classified into macro-, mini- and microsatellites. Macrosatellites, or satellites, span up to hundreds of kilobases within the constitutive heterochromatin. They differ substantially from the rest of the genome in nucleotide content and hence can be separated by buoyant density gradient centrifugation, as satellite bands.^11^ An example of a macrosatellite element is the *α* satellite family discussed below. Minisatellite arrays are somewhat shorter. For example, telomeric repeats with a short hexanucleotide repeat unit located at chromosomal ends span 10–15 kilobases in humans. Microsatellites are the smallest tandem repeats, and among the most variable DNA sequences.^12^ The most common CA/TG dinucleotide tandem repeats constitute 0.5% of the human genome.

## REPEAT-DERIVED ncRNAs, repRNAs

Rapid advances in next-generation sequencing allowed a deep insight into transcriptomes, and the ENCODE consortium reported that highly repetitive genomic regions are also transcribed in humans. These reports opened a lively debate about potential functions of these transcripts. The widespread transcription of repetitive DNA can (1) produce functional, active ncRNAs, (2) be important per se to set the chromatin state or to interfere with transcription of other genes, or (3) simply be an insignificant background process. There is no straightforward way to distinguish between meaningful transcripts and transcriptional noise. So far, evolutionary conservation served as a good indication of RNA function. However, recently this correlation has been under debate.^13^–^15^ At this moment, only the analysis of individual RNAs can yield data on their functionality.

**Figure 2:**
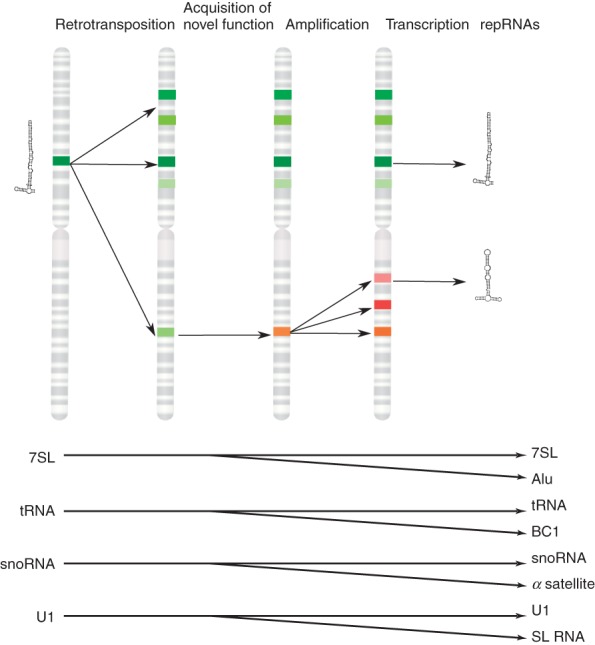
Repeat-derived RNAs (repRNAs) often originate from retrotransposed non-coding RNAs (ncRNAs). Top: Upon retrotransposition, ncRNAs are highly amplified, and as they spread throughout the genome, they diversify in sequence (depicted as bands of different shades of the same color). Some copies evolve new functions (depicted as a band with a changed color) giving rise to new classes of repRNAs. Therefore, non-coding transcripts derived from highly repetitive regions can be a rich reservoir for the evolution of novel functional RNAs. Bottom: Examples of repRNAs and their corresponding ancestor mastergenes. For detailed discussion, see text.

The impact of repeats on the evolution of genomes and protein-coding genes has been described elsewhere.^4^,^16^ Here, we summarize what is known about the evolution and function of several ncRNAs expressed from repetitive DNA. We coin the term repRNAs (repeat-derived RNAs) for non-coding transcripts with a distinct activity, which are expressed from repetitive elements. We present a hypothesis that functional repRNAs can originate from retrotransposon-propagated ncRNAs. By acquiring the ability to retrotranspose, ncRNAs can become highly amplified and spread throughout the genome. Some of the new copies escape previous evolutionary constraints, accumulate mutations, and as a result lose their original function and might acquire novel activities. Therefore, transcripts derived from highly repetitive regions can be a rich reservoir for the evolution of novel functional RNAs (Figure 2). It has to be kept in mind that even if a repRNA evolves new activities, it does not necessarily bring about a functional change in the cell. Only if the novel activity leads to a downstream cellular event, we can clearly attribute a function to these novel ncRNAs.

## EXAMPLES OF repRNAs EVOLVED FROM ncRNA

### Signal Recognition Particle 7SL RNA as the Ancestor of Alu Elements

Alu repeats are a primate-specific SINE family. They are approximately 300 bp in length and originated from a ncRNA, the signal recognition particle component 7SL RNA, through processing and duplication.^17^,^18^ Alu and its rodent counterpart B1 RNA evolved from 7SL in a common ancestor of primates and rodents around 100 million years ago.^19^,^20^ There are approximately 10^6^ copies of Alu elements making up 10.7% of the human genome. Similarly, there can be up to 10^6^ B1 elements in rodent genomes.^21^ The 7SL RNA is the first representative for our model of retrotransposon-mediated evolution of novel RNAs: the 7SL RNA was retrotransposed, then propagated to a very high copy number to eventually give rise to ncRNAs with novel activities as well as several RNA domains that impact on gene evolution and expression.

Because SINEs contain an original RNA polymerase III promoter, Alu elements can be transcribed into individual RNAs. They have been shown to be induced in stress conditions, such as heat shock or cycloheximide treatment,^22^ and to inhibit transcription of RNA polymerase II in *trans*.^23^ It has been proposed that direct interaction of Alu and RNA polymerase II at promoters leads to down-regulation of housekeeping transcription, presumably as a part of complex cellular stress response.^23^ If this novel activity of Alu ncRNA has a functional relevance for the cell, this still needs to be demonstrated.

Alu sequences are also present as domains embedded in many transcripts of protein-coding genes, as well. The Alu consensus sequence contains up to 10 potential 5′ donor splice sites and up to 13 potential 3′ acceptor sites.^24^ As a consequence of many Alu insertions into genes, 5% of all alternatively spliced exons within protein-coding regions contain Alu sites. Thus, Alu sequences are elements that play an important role in the evolution of novel genes. An interesting example was reported where an Alu element gave rise to a novel 5′ exon in the human tumor necrosis factor type 2 gene (*p75TNFR*), providing a novel N-terminal protein domain resulting in a novel receptor isoform.^25^ In addition, gene-integrated Alus can be a source of promoters, enhancers, silencers, insulators and influence mRNA stability.^26^

Thus, 7SL is a prominent example of an ncRNA that has evolved diverse functions upon retrotransposition and amplification. The second lineage of SINEs derived from 7SL, the B1 elements, is much less studied than the Alu elements, but there is evidence that it has also evolved regulatory functions in rodents.^27^

### tRNA-Derived ncRNAs

LINE-1 reverse transcriptase is thought to recognize LINE-1 mRNA partially by a sequence-specific fashion and partially by a mechanism called *cis*-preference. While the RT is being translated, the nascent protein simply binds the nearest RNA, which most often is the mRNA that encodes it.^28^ In order for SINEs to exploit *cis*-preference and serve as template for LINE-1 RT, they have to be able to come close to the translating ribosome.^26^ Therefore, it comes as no surprise that the vast majority (96%) of SINE families originate from tRNAs.^29^,^30^

tRNAs have evolved diverse functions after retrotransposition and amplification. Rodent-specific neuronal BC1 RNA is a translational repressor that specifically targets eIF4A and strongly impedes its helicase activity.^31^ BC1 is 152-nucleotide long, twice the length of tRNA^Ala^. While the sequence similarity of mouse tRNA^Ala^ and the BC1 5′ region amounts to 80%, the secondary structure is a stable hairpin instead of a cloverleaf-like structure. The BC1 gene was generated by retrotransposition of tRNA^Ala^ and arose after the mammalian radiation but before the diversification of Rodentia. The cDNA copy of tRNA^Ala^ was integrated in a locus that is expressed specifically in neurons.^32^,^33^

Another example of tRNA SINE-derived functional RNA is B2, which is present on average in 10^5^ copies throughout rodent genomes.^34^ The heat shock-induced B2 is transcribed by RNA polymerase III into RNAs of variable sizes from 200 to 600 nucleotides.^35^ B2 consists of the 5′ tRNA-like sequence^36^ followed by a polyadenylated 3′ tail.^37^ Rodent B2, like human Alu, was proposed to be a specific inhibitor of RNA polymerase II, binding an RNA-docking site in the core polymerase complex and, as a consequence, preventing the formation of an active closed complex.^38^,^39^ Espinoza et al. 40 further showed that a 51-nucleotide sequence of the B2 3′ region was responsible for repressing RNA polymerase II activity.

### snoRNAs Are Ancestors of *α* Satellite RNAs

The primate-specific *α* satellites belong to long tandem repeats and consist of 171-bp-long units organized in a head-to-tail manner. Human *α* satellites are annotated at 44,058 loci covering 0.1% of the genome. Each human centromere contains a chromosome-specific higher-order array of *α* satellites^41^ that are positioned tandemly to span 3–5 Mb. Typically, the units within the higher-order repeats are highly similar (95–100% identity)^42^,^43^ due to sequence homogenization. In the pericentromeric regions, *α* satellites occur as monomers that are often intermingled by other repeats, such as SINEs, LINEs, LTRs or β satellites. Interestingly, the sequence similarity shared by those monomers is much lower than that of the units within higher-order repeats. In addition, comparative sequence analyses reveal that the sequence of *α* satellite paralogues within higher-order repeats differs substantially less than *α* satellite orthologs among primates.^44^ All of those observations, together with the fact that centromeres of ‘lower’ primates consist of *α* satellite monomers, are the basis for the hypothesis that initial higher-order arrays of *α* satellites originated from the progenitor monomeric sequence that was transposed and propagated in chromosomes of ‘higher’ primates forming functional centromeres.^44^,^45^

We have proposed snoRNAs as ancestors of human *α* satellites (Matylla-Kulinska et al., unpublished). The predicted secondary structure of the consensus sequences of human *α* satellite families retrieved from the Dfam database^46^ resembles the structure of H/ACA-snoRNAs. It contains two stems joined by an unstructured linker enclosing degenerated H- and ACA-boxes (Matylla-Kulinska et al., unpublished). The evolutionary most distant homologs to human *α* satellites were identified in marmosets.^47^ The structural analysis of marmoset alphoid sequences revealed a degenerated snoRNA-like structure. Interestingly, the consensus fold comprises a 3′ flank region similar to the one previously characterized in marsupial snoRNA-derived retrotransposon, snoRTEs.^48^ SnoRTEs including H/ACA snoRNA combined with retrotransposon-like non-LTR transposable elements (RTEs) were reported to have an ability to insert into new genomic loci. In addition, dyskerin, which is a centromere-binding factor 5 (Cbfp5) homolog and a core member of H/ACA snoRNPs, seems to be also involved in mitotic spindle formation and in the spindle assembly checkpoint.^49^ Our structural bioinformatic data together with the above-mentioned observations point to snoRNAs as primary sequence origin for primate *α* satellites.

In the course of mutation accumulation, segment duplications and sequence conversion, *α* satellites lost a snoRNA-related function, but their centromeric location allowed them to acquire some new functions instead. It is well established that the centromere and the underlying DNA are important for the following: (1) recognition and pairing of homologous chromosomes, (2) coupling of the sister chromatids during nuclear division, then either releasing the joint (during mitosis and second meiotic division) or retaining it (first part of meiosis), as well as (3) the spindle formation.^50^–^52^ Moreover, *α* satellites function also on the RNA level, as the *α* satellite transcripts are crucial for proper localization of centromere-specific proteins CENP-C1 and INCENP.^53^ Results obtained in our laboratory (Matylla-Kulinska et al., unpublished) indicate that *α* satellite-derived aptamers can not only bind to Pol II but also serve as templates for RNA-dependent RNA polymerization and/or 3′ extension, both catalyzed by RNA polymerase II. However, the function of this interaction needs to be further elucidated.

### U1 small nuclear RNA Evolved into Spliced Leader RNA Multiple Times

In addition to *cis*-splicing, i.e., the removal of introns from pre-mRNAs, some phylogenetically distant organisms employ *trans*-splicing during mRNA biogenesis. In *trans*-splicing, the 5′ portion of a pre-mRNA is substituted with a spliced leader RNA (SL RNA), which is transcribed from a distinct genomic locus. As a consequence, many mRNAs (in some organisms all mRNAs) share a common 5′ end (reviewed in Ref 54). *Trans*-splicing can have a multitude of functions, e.g., processing of polycistronic pre-mRNAs into individual mature mRNAs, providing 5′ cap structure and thereby stabilizing the transcript, and providing initiator AUG codon.^54^,^55^

There is evidence that SL RNAs evolved from the repetitive spliceosomal U1 small nuclear RNAs (snRNAs). Both RNA classes possess a trimethylguanosine cap structure and Sm-binding site; they are often dispersed in arrays of 5S rDNA, and the *trans*-splicing machinery utilizes other snRNA components of the major spliceosome except U1. Indeed, it has been shown that SL RNA can complement U1 loss in an *in vitro* splicing system.^56^ These similarities made it possible for SL RNAs to evolve independently several times in distant eukaryotic species.^57^,^58^

U1 and other snRNAs behave like TEs, giving rise to large families of pseudogenes.^59^ It has been suggested that some of the pseudogene families are in fact the ancestral form of U1, indicating that U1 itself is an ncRNA derived from repeat elements.^60^ During the evolution of eukaryotes, some of the U1 elements invaded the 5S rDNA repeat unit and became a part of a large array.^61^,^62^ SL RNAs might have evolved from these 5S rDNA-linked U1 elements, but perhaps they retained the capability to transpose, as they have been found dispersed at other genomic loci as well. We envision that SL RNAs and U1 snRNAs still have the ability to give rise to functionally distinct RNAs, as some U1 paralogs have been shown to be differentially expressed and are reported to have tissue- and developmental stage-specific functions.^63^,^64^

## CROSS-ANALYSIS BETWEEN Dfam AND Rfam IMPLIES MANY MORE EXAMPLES OF repRNAs

**Figure 3:**
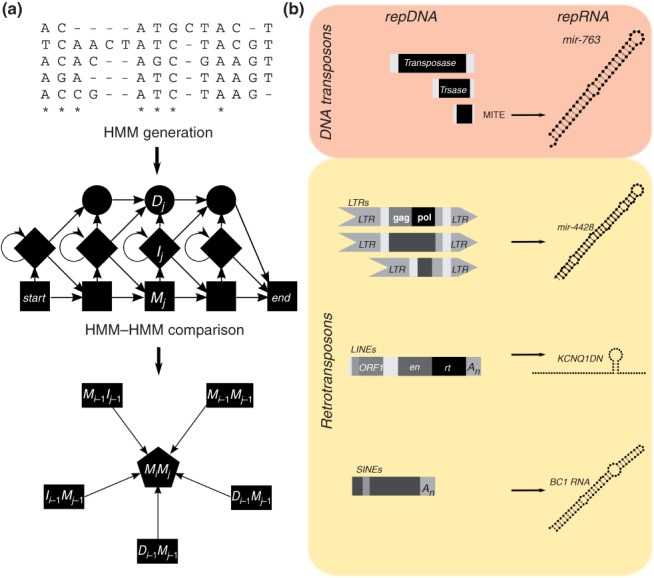
Comparison of Dfam with Rfam reveals new relationships between repeat elements and non-coding RNAs (ncRNAs). (a) For each repeat and ncRNA family found in Dfam and Rfam, respectively, an hidden Markov model (HMM) was constructed based on the corresponding seed alignments. These HMMs were then compared by literally aligning the states of both HMMs using dynamic programming. The best state alignment ending with the alignment of match state *M_i_* and *M_j_* can be obtained either from *M_i−1_M_j−1_*, *D_i−1_M_j−1_*, *M_i−1_D_j−1_*, *M_i−1_I_j−1_* or from *I_i−1_M_j−1_*. (b) Examples of novel relationships between repeat elements and ncRNAs. mir-763 shows strong similarity with a MITE, mir-4428 derives from long terminal repeats (LTRs). KCNQ1DN ncRNA is highly homologous to long interspersed nuclear elements (LINEs).

In order to investigate whether there are other ncRNAs derived from repeat elements, we took a systematic approach to assess sequence similarity between the repeat families found in Dfam^46^ and the ncRNA families found in Rfam.^65^ To this end, hidden Markov models (HMMs) were generated from the seed alignments of the corresponding Dfam/Rfam entries as well as the MirBase miRNAs with the help of the HMMER packages.^66^ These HMMs were then compared based on an own implementation of the algorithm published in Ref 67, taking special interest in RNAs. The HMM–HMM comparison can be conceptualized as an alignment of HMM states. The corresponding scoring function takes into account the transition probabilities of the HMMs and the emission probabilities along the HMMs at the same time (see Figure 3(a)). This approach was chosen to improve the sensitivity and speed of the search as well as to facilitate the homology scoring by returning a single score and significance value for each HMM comparison. In order to assess the significance of the HMM comparison, a score distribution was computed for each Dfam HMM model. This was done by approximate dinucleotide shuffling 10 times the seed alignments used to generate the HMMs and generating the HMMs for each of the shuffled alignments, leading to a total of 11,320 HMMs. For each Dfam HMM, the score distribution was then fitted by a Gumbel extreme value distribution in order to compute the significance value directly from the HMM–HMM comparison score.

The outcome of our cross-analysis unambiguously shows that the strong similarity between ncRNAs and RNAs derived from human repeats is predominantly seen for miRNAs. From the 1433 ncRNAs having a *P*-value smaller than 10^–5^, a threshold that corresponds to the previously reported sequence similarity between the mir-325 family and the L2 repeats,^68^ 87% (1248) were related to miRNAs. The vast majority of the miRNAs are homologous to Alu elements (SINEs), followed by LINEs, DNA transposons, and LTR as reviewed in Ref 69. Furthermore, we found a complete overlap between the 3′ end of LFSINE_vert and uc_338 (ultraconserved element) confirming a previous report from Refs 70 and 71, and high similarity between the central region of Plat_L3 and imprinted long ncRNA, KCNQ1DN. Our analysis also confirms reports on other homologies, such as BC200 and 7SL. Then, we scanned the genomes of mouse, platypus, and chicken with a similar approach. In order to generate the repeat-HMMs, the RepeatMasker annotation of the corresponding genomes was downloaded from the UCSC genome browser^72^ and was used to generate alignment for each repeat family. These alignments were passed to HMMER in order to generate the repeat HMM. Similar to the results of human analysis, the majority of the repRNAs from mouse, platypus, and chicken are miRNAs derived from DNA repeats and LINE elements. In contrast, no similarity between SINE elements and uc_338 could be found. In the lizard *Anolis carolinensis*, however, similarity between uc_338 and LFSINE_vert was detected. We also identified mir-7641 as a derivative of rRNA repeats, as well as mir-763 and mir-1641, which derive from DNA repeats. For complete results, see http://alu.abc.univie.ac.at/reprna.

## SEARCHING FOR FUNCTIONS OF repRNAs

The protein-coding parts of genomes are thoroughly investigated, but very little attention is brought to the large quantity of sequences that are not unique and do not belong to the conventional concept of a gene. Poor interest in repetitive arrays arises in part from the following two reasons: they are considered to be ‘junk’ or non-functional, and their repetitive nature hampers the computational annotation and analysis of those parts of the genome. Canonical genetic and biochemical methods cannot easily be applied to address the function of highly repetitive elements. Yet it became obvious that repeat regions are not silent, but differentially expressed in various states of the cells.^73^

In order to look for repRNA functions, biochemical and bioinformatic approaches are necessary. We recently employed Genomic SELEX combined with deep sequencing as an unbiased approach to screen entire genomes for short functional RNA motifs that bind to specific ligands of choice.^74^ It is feasible to examine whole genomes because RNA libraries used for this approach are transcribed *in vitro* from genomic DNA and hence contain all potentially functional domains encoded in a genome regardless of their expression levels. Importantly, in these genomic libraries, the repeat-derived sequences are equally represented compared with genic sequences, making the approach especially suitable for the analysis of repRNAs. The limitation of SELEX screens is the choice of baits that are used to isolate the target RNAs. On the other hand, once a protein–RNA interaction is detected, the protein will deliver first hints on the functionality of the RNA.

## CONCLUSION

We showed that repRNAs, derived from ncRNAs by retrotransposition and amplification, are a potent source of new functional RNAs. We illustrated the phenomenon with four examples, but it is likely that there are more ncRNAs that evolved new functions after retrotransposition. Sequence conservation across species may suggest function. Thus, additional repRNAs might be derived, for instance, from conserved SINE descendants, 4.5S_I_ and 4.5SH RNAs.^75^,^76^ Similarly, interaction of ncRNA with a cellular protein might imply function, as can be the case of snaR family.^77^

It is important to note that repRNAs (and thus the evolutionary reservoir) can arise by different mechanisms as exemplified by telomeric TERRA RNA. TERRA transcripts are products of RNA polymerase II, but the telomeric loci are produced by the telomerase enzyme, which solves the end-replication problem. Telomerases extend telomeric 3′ ends through reverse transcription using short telomere RNA as template.^78^,^79^ This template contains a short sequence, which is copied in a repetitive fashion, leading to an array containing many short tandem repeats. Telomerase-like reverse transcription is an example of how long tandem repeats can originate.

Similarly, not only origins of repRNAs are diverse, so are newly evolved functions and mechanisms of action, which do not necessarily remain on the RNA level. For example, RNA polymerase III-transcribed genes are generally repetitive,^80^ and in many loci of various genomes, the coding sequence has been lost and ‘orphan’ RNA polymerase III promoter elements play a role, for instance, in the regulation of RNA polymerase II transcription^81^ and possibly also in chromosome organization.^82^ Similarly, tRNA genes, a class of RNA polymerase III transcripts, have been shown to regulate the expression of neighboring RNA polymerase II genes^83^ or act as chromatin insulators.^84^

The evolution of new functions of repRNAs can be hindered by a process of concerted evolution in which gene conversion or unequal crossover leads to overwriting of a repeat with the sequence of its paralog, and the repeats are thereby homogenized in a given genome. The phenomenon is documented in repeats arranged in arrays, for instance in rDNA and *α* satellites,^85^,^86^ and is beneficial when a gene product is needed in great abundance, as is the case of rRNAs and histone mRNAs.^87^ Nevertheless, whether other gene families undergo concerted evolution is questionable,^88^ and many of them clearly diverged to the point where gene conversion is no longer possible.

Repeat elements have long been ignored in genomic annotation and high-throughput data analyses. Nevertheless, this is changing due to the recognition of their importance for genomes and transcriptomes. We can therefore expect that many more functional repRNAs will be discovered in future research.
